# Electrical Conductivity of Rubber Composites with Varying Crosslink Density under Cyclic Deformation

**DOI:** 10.3390/polym14173640

**Published:** 2022-09-02

**Authors:** Hamed Peidayesh, Zdenko Špitalský, Ivan Chodák

**Affiliations:** Polymer Institute of the Slovak Academy of Sciences, Dúbravská Cesta 9, 845 41 Bratislava, Slovakia

**Keywords:** electroconductive rubber composite, mechanical deformation, vulcanization time, crosslink density, conductive pathway

## Abstract

Studies addressing electroconductive composites based on rubber have attracted great interest for many engineering applications. To contribute to obtaining useful materials with reproducible behavior, this study focused on understanding the mechanism of conductivity changes during mechanical deformation for rubber composites based on styrene-butadiene rubber (SBR) or ethylene-propylene-diene terpolymer (EPDM) vulcanized for various times. The composites were characterized by static electrical conductivity, tensile testing, dynamic mechanical thermal analysis (DMTA), and crosslink density measurements. The tensile strength and Young’s modulus were found to increase significantly with rising vulcanization time. Higher static conductivity values of the composites were observed with the increase in vulcanization time. The most important aspect of this investigation consisted in the electrical current measurement online with recording the stress-strain curves, revealing the details of the uniaxial cyclic deformation effect on changes in the structure of conductive pathways indirectly. The electrical conductivity during five runs of repeated cyclic mechanical deformations for SBR composites increased permanently, although not linearly, whereas EPDM composites showed a slight increase or at least a nearly constant current, indicating healing of minor defects in the conductive pathways or the formation of new conductive pathways.

## 1. Introduction

Electrically conducting polymer composites are obtained by mixing conducting fillers in an insulating polymer matrix. Conventional conducting fillers are micrometer- or submicrometer-scale metal powders or carbonaceous fillers e.g., carbon black (CB), graphite, or carbon fibers [1,2]. In order to achieve high conductivity similar to semiconductors or even conductors, the filler content needs to be as high or higher than the so-called percolation threshold, which is defined as a narrow concentration range of the filler in which the electrical conductivity increases by several orders of magnitude [3]. The principle of such conductivity increase consists in a formation of an electroconductive physical network through the whole cross section of the specimen, enabling facile and fast transport of electrons within the polymer matrix. During the last decade, these conducting composites have been considered to be relatively cost-effective materials for many engineering applications, such as sensors, antistatic coatings and films, conducting adhesives, and electromagnetic interference shielding materials [4,5,6].

The conductivity of these composites depends on several parameters, such as intrinsic properties, state of the dispersion, and the geometry of the nanofillers, as well as the filler-matrix interactions. Furthermore, for any possible application, the intimate relationship between the conductivity changes and external stimuli, such as electrical, thermal, mechanical, and chemical stresses, must be understood in detail [7]. From this point of view, mechanical deformation can be expected to be the most important factor affecting the structure of the conducting paths and eventually leading to substantial destruction of the filler network structure. Such a situation is caused by numerous factors, the most deterministic of which are the type and composition of components, their adhesion and cohesion, and the method of composite preparation.

Despite a number of relevant papers investigating the electrical and mechanical properties of electroconductive composites, studies addressing the dependence of electrical conductivity on mechanical deformation using online measurements seem to be of particular interest. In this case, electrical conductivity can indirectly define the structural features of the filler topology, indicating whether the conductive pathways remain intact, are destroyed, reformed, or new ones are formed [2,3]. Aneli et al. [8] and Flandin et al. [9] used this approach to detect the changes in electrical conductivity during deformation. Various vulcanized rubbers filled with different types of carbon black were evaluated, either in tensile or in compression mode. In addition to the uniaxial deformation with varying strains, other treatments, such as cyclic deformation and mechanical relaxation, were studied, which indicated the instant changes of conducting pathways and reinforcing structures affecting the stress-strain curves. The method was described and discussed in detail by us [2,10,11], providing clear correspondence of the changes in electrical conductivity with features of stress-strain curves for the composite filled with CB particles, as well as describing the instant changes of conducting pathways during mechanical deformation, not only for vulcanized rubber matrices but also for thermoplastics. The effects in the two different matrices were compared and important similarities were observed [3].

Investigating both electrical conductivity and mechanical properties in parallel using online measurements is described in the literature [8,12]. However, to date, no investigation has been done to evaluate the effect of vulcanization time on the electrical conductivity of styrene-butadiene rubber (SBR) and ethylene-propylene-diene terpolymer (EPDM) rubber composites under mechanical tensions. The main goal of the present work was to investigate the influence of various curing times on the electrical conductivity behavior of the rubber composites based on SBR and EPDM during uniaxial mechanical deformation. In this regard, the physical and mechanical properties and static conductivity were evaluated. The main novelty of the research consists in the comparison of the stress-strain curves and the changes in electrical conductivity under cyclic deformation. As far as we are concerned, parallel measurements of mechanical properties and electrical conductivity have not been measured in cyclic mode up to now.

## 2. Materials and Methods

### 2.1. Materials

Styrene-butadiene rubber (SBR, SKS 30, KAUČUK Kralupy a.s., Kralupy nad Vltavou, Czech Republic) and ethylene propylene diene terpolymer (EPDM, KEP–281F, DSM elastomers) were used as rubber matrices. Carbon black (Vulcan^®^ N–234, Cabot Corp., Lešná, Czech Republic) was used as the standard highly conductive filler. All the properties of this material provided by the producer are summarized in Table 1. A vulcanizing system consisted of N-cyclohexyl-2-benzothiazole sulfenamide CBS (Duslo, Šaľa, Slovakia) as the accelerator, stearic acid (Setuza, Ústí nad Labem, Czech Republic) and zinc oxide (Slovlak, Košeca, Slovakia) as activators, and sulfur (Siarkopol, Tarnobrzeg, Poland) as curing agent were supplied.

### 2.2. Mixing, Calendaring, and Curing Process of Rubber Composites

The rubber composites were prepared by two-step mixing of rubber matrices, CB particles, and vulcanizing system in a 50 mL laboratory mixer (Plastograph PLE 331, Brabender, Duisburg, Germany). The rubber, filler, and activators (ZnO and stearic acid) were compounded in the first step for 10 min at 100 °C and 40 rpm, whereas the accelerator (CBS) and sulfur were introduced in the second step for 8 min at 90 °C and 25 rpm. The compounds were further calendered to increase the homogeneity using a laboratory two-roll mill (Nishimura, Tokyo, Japan). The calendering process was conducted through sheeting, rolling, and sheeting steps at room temperature. Two rubber compounds were prepared with the same ratio of sulfur and other ingredients to rubber, while the amount of CB was kept constant at 70 phr or 40 wt%. The composition of rubber compounds is summarized in Table 2.

The curing process was performed by compression molding using a laboratory press (Fontijne TP 50, Delft, The Netherlands) at 150 °C for various curing times of 3, 8, 30, and 60 min. The samples were marked as SBR-X and EPDM-X, where X is the time of curing. To prepare the specimens for online measurements of the electrical current during mechanical deformation, two copper wires were inserted into the material during compression. The specimens (0.7 mm thick) with dimensions of 100 × 10 mm were compression molded using a custom mold. The specimens were kept in PE bags for 24 h before deformation.

### 2.3. Mechanical Properties and Dynamic Mechanical Thermal Analysis (DMTA)

Dumbbell-shaped test specimens (width 3.5 mm, deformed length 30 mm, and thickness of approximately 1 mm) were obtained by punching the compression-molded slabs using a pneumatic toggle press. The tensile properties of rubber composites were measured using an Instron 3365 universal testing machine (Instron, Norwood, MA, USA) in uniaxial deformation at cross-head speed of 50 mm·min^−1^. The mean values and standard deviations were calculated from seven specimens for all parameters.

Dynamic mechanical performances of the rubber composites were evaluated using the instrument DMA Q800 (TA Instruments, Hüllhorst, Germany). The specimens (ca. 10 × 7 × 1 mm^3^) were measured in tensile mode at a frequency of 10 Hz and an amplitude of dynamic deformation of 20 μm. The experiments were conducted in the temperature range of −60 °C to 100 °C with a heating rate of 2 °C·min^−1^.

### 2.4. Electrical Conductivity

A Concept 40 (Novocontrol) was applied for broadband dielectric spectroscopy. For this purpose, an instrument with an Alpha dielectric spectrometer (Novocontrol Technologies GmbH, Montabaur, Germany) was used. The specimens for the dielectric spectroscopy were of disk shape with a diameter of 20 mm and a thickness of ~0.5 mm. Electrical conductivity was determined in the frequency range of 0.1 Hz to 1 MHz at room temperature. As a test cell, the BDS–1200 parallel-plate capacitor with two gold-plate electrodes was used.

### 2.5. Online Measurements of Conductivity during Cyclic Mechanical Deformation

**Scheme 1:**
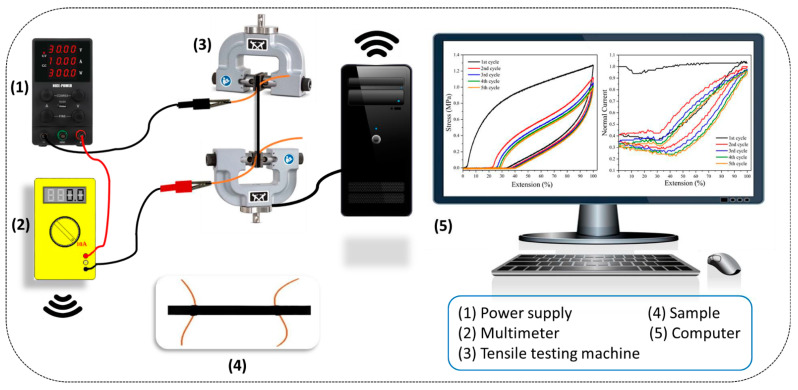
Setup for online measurement of electrical conductivity changes during cyclic mechanical deformation [2].

A wireless TRMS multimeter (EXTECH Instruments, Shanghai, China) was used to record the electrical current in parallel with recording the stress-strain curve. The procedure for sample preparation and the machine for mechanical deformation are described in Section 2.2 and Section 2.3, respectively. The conductivity measurement was carried out up to ultimate extensions of 100% during five cycles of deformation. The voltage of power supply and the rates of deformation were set to 30 V and 10 mm·min^−1^, respectively. Scheme 1 shows the setup for online measurement of conductivity changes during cyclic deformation [2].

### 2.6. Solvent Uptake and Crosslink Density

To determine the crosslink density of rubber, the equilibrium swelling of samples in proper solvent was performed according to the ASTM D6814 standard. Triplicate specimens of each composite were swelled in toluene until equilibrium at ambient temperature and 48 h. The specimens were taken out from the solvent and weighed after the removal of toluene from the surface. Then, the samples were dried in an oven at 120 °C for 6 h until a constant weight was reached. The swelling degree of the rubber composites was determined using the gravimetric method by Equation (1):

(1) $$\mathrm{Solvent}\;\mathrm{uptake}\;(\%)=\frac{m-m_{0}}{m_{0}}\times 100$$

where *m*_0_ and *m* refer to the sample weight before and after swelling, respectively.

The Flory-Rehner equation (Equation (2)) was applied to calculate the crosslink density, *ν*, by using the obtained equilibrium swelling measurements [13].

(2) $$-\ln\left(1-v_{r}\right)-v_{r}-\chi v_{r}^{2}=2\rho v_{s}vv_{r}^{\frac{1}{3}}$$

where *ρ* is the rubber density, *ν_r_* is the volume fraction of swollen rubber, *χ* is Huggins rubber-solvent interaction parameter (for EPDM-toluene *χ* = 0.496 [14], for SBR-toluene *χ* = 0.31 [15]), and *ν_s_* is the molar volume of solvent (for toluene *ν_s_* = 106.3 mL·mol^−1^). The *ν_r_* is given by Equation (3):

(3) $$v_{r}=\frac{\left(\frac{w_{d}}{\rho_{r}}\right)}{\left(\frac{w_{d}}{\rho_{r}}\right)+\left(\frac{w_{s}-w_{d}}{\rho_{s}}\right)}$$

where *w_d_* and *w_s_* are the weight of dry and swollen samples, respectively, and *ρ_s_* and *ρ_r_* are the density of the solvent (for toluene *ρ_s_ =* 0.867 g·cm^−3^) and the rubber, respectively.

## 3. Results and Discussion

### 3.1. Mechanical Properties

**Figure 1:**
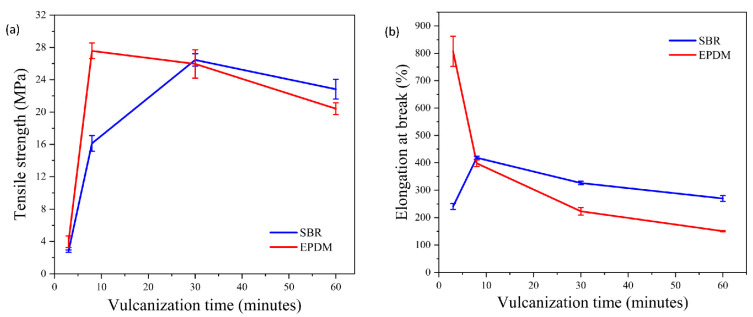
Dependence of (**a**) tensile strength and (**b**) elongation at break of composites as a function of vulcanization time.

The values of mechanical properties, including tensile stress, elongation at break, Young’s modulus, and moduli M100, M200, and M300 for vulcanizates of EPDM and SBR are summarized in Table 3. Figure 1 presents the dependence of tensile strength and elongation at break of composites as a function of vulcanization time. It is seen that the tensile strength of the composites increases with rising vulcanization time up to an optimum (in our case, 8 and 30 min at 150 °C for EPDM and SBR composite, respectively), while further increase in curing time results in a slight decrease in the tensile strength. The SBR composite shows a substantial enhancement in tensile strength from 2.81 to 26.45 MPa, while Young’s modulus is increased from 7.28 to 11.39 MPa. Moreover, the EPDM composite presents an increase in tensile strength from 3.97 to 27.57 MPa and a maximum Young’s modulus of 16.26 MPa. As seen in Table 3 and Figure 1, the elongation at break decreases with rising vulcanization time.

**Figure 2:**
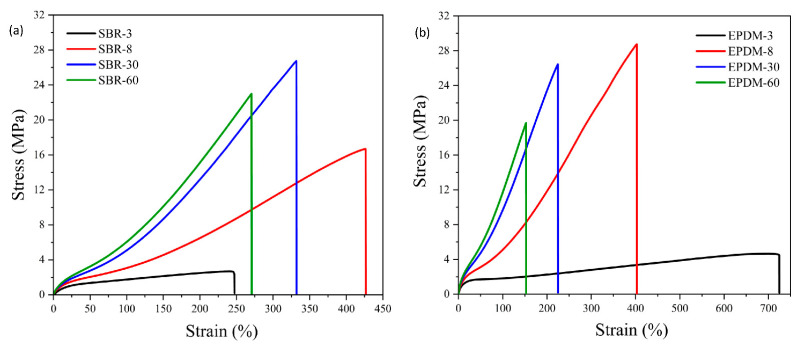
Stress-strain curves for (**a**) SBR and (**b**) EPDM composites vulcanized for various times. In the images, the vulcanization time at 150 °C is shown in minutes as the number after code SBR- or EPDM-.

Basically, the vulcanization system and type of rubber determine the level of crosslink density. Thus, more homogeneous crosslink distribution along the elastomer chains leads to a stronger network, resulting in higher tensile characteristics and lower elongation at break. On the other hand, the restriction in mobility and orientation of macromolecular chains caused by crosslinks should be considered the main reason to explain the decrease in tensile strength [16,17]. This explanation is supported by the shape of the stress-strain curves for the composites displayed in Figure 2. Moreover, it becomes apparent that the formation of the crosslink between EPDM and sulfur occurs in a shorter time compared to the SBR vulcanizate. A possible explanation for this phenomenon might be the existence of very reactive monomer units in EPDM rubber, which are distributed randomly along the rubber chains, leading to a fast reaction in sulfur vulcanization [18]. All these findings indicate the relationship between the mechanical properties and the degree of crosslinking, at least for the dependence of curing time on the tensile strength, elongation at break, and Young’s modulus.

### 3.2. Solvent Uptake and Crosslink Density

The swelling degree of rubber vulcanizates by selected organic solvents is routinely used to determine crosslink density. Solvent uptake is affected by several factors, such as temperature, type of solvent, and strength of thermodynamic interactions between the solvent molecules and polymer chains [15,19] and especially the average chain length between the crosslink junctions. The swelling degree and corresponding crosslink density of SBR and EPDM composites are presented in Table 4. The results reveal that the solvent uptake of vulcanizates decreases with the increase in the crosslink density caused by rising the time of curing. Obviously, the reason for such behavior is an increase in rigidity and matrix chain mobility decrease during vulcanization. Theoretically, the elastically active linkages are formed within the macromolecular structure of the rubber by the sulfur crosslinking system. These linkages are monosulfides, disulfides, and polysulfides, as well as cyclic linkages that only give intramolecular bonds during the vulcanization process. Monosulfide linkages produce maximum restriction in the chain mobility of rubber due to shorter bridge links in C–S_x_–C [20]. Therefore, the obtained results from the swelling studies are in agreement with the mechanical properties.

### 3.3. Dynamic Mechanical Thermal Analysis (DMTA)

**Figure 3:**
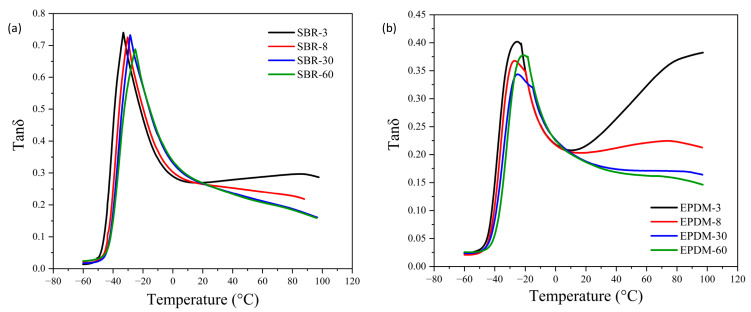
Tanδ curves for (**a**) SBR and (**b**) EPDM composites vulcanized for various times. In the images, the vulcanization time at 150 °C is shown in minutes as the number after code SBR- or EPDM-.

DMTA analysis was performed to evaluate the viscoelastic response of the rubber composites associated with the macromolecular motions and internal changes in the structure. The data of tanδ of the composites as a function of temperature is shown in Figure 3. The dependence of the glass transition temperature (T_g_) on the crosslink density of the composites is also shown in Table 5. It is seen that with rising time of curing and consequently, increasing the crosslink density, the T_g_ shifts to higher temperatures. This behavior indicates rigidity increase and lower molecular mobility due to crosslinking reaction. It is known that several factors, such as crosslink density, chain rigidity, and the type of crosslinking system, can affect the T_g_ values of the crosslinked sample [16,21,22]. Considering the type of rubber matrix, it is seen that the T_g_ of SBR composites is lower compared to EPDM due to the lower crosslink density and possibly also different microstructure of these composites.

An interesting feature is seen for the curve shapes at temperatures above 60 °C, consisting in an increase of tanδ values for samples vulcanized for shorter times, while a decrease was observed in cases of longer vulcanization times. Moreover, the effect is much more pronounced for EPDM than for SBR.

Generally, the tanδ values are calculated as E″/E′, where E″ is the loss modulus and E′ is the storage modulus related to Young’s modulus. The loss modulus is related to energetic losses due to increased mobility of rubber chains, while E′ reflects the stiffness of the material. The crosslink density of EPDM indicates that after 3 min of curing, the material is undervulcanized, corresponding to low tensile strength and extremely high elongation at break (compared to strength and elongation after longer vulcanization times). With rising temperature during DMTA measurement, the mobility of the chains is increasing while a decrease of storage modulus should be expected; therefore, tanδ values are rising. However, the difference in crosslink density between vulcanization times 3 and 8 min is very high, so it may be expected that not only E′ will increase but also the increase of E″ will be much lower, if any. Therefore, the effect of extension of the curing time leads to the decrease in the tanδ values.

Although the SBR shows similar behavior as the EPDM, the absolute changes are much smaller. The differences may consist in the higher absolute values of tanδ for SBR at Tg, so that relative increase-decrease at around 60 °C is smoother, but also a possible certain degree of thermal degradation of SBR during long vulcanization times, which may affect not only the crosslinking density but also the most stressed rubber segments for which the effect on the mobility is largest. These sites are of much lower importance in thermally substantially more stable EPDM.

### 3.4. Electrical Conductivity

**Figure 4:**
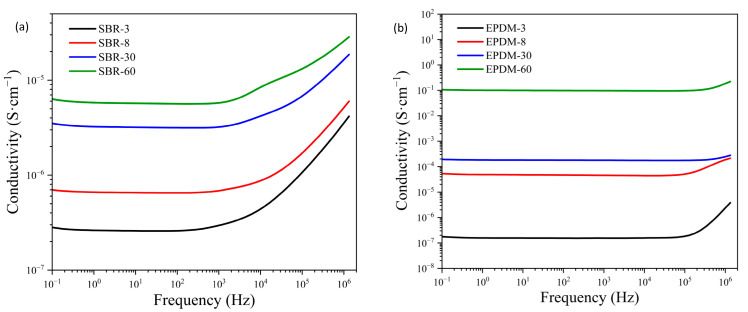
Electrical conductivity of (**a**) SBR and (**b**) EPDM composites vulcanized for various times as a function of frequency. In the images, the vulcanization time at 150 °C is shown in minutes as the number after code SBR- or EPDM-.

The conductivity of SBR and EPDM composites under static conditions were measured to investigate the effects of vulcanization time on the electrical conductivity of composites. Figure 4 shows the frequency-dependent conductivity of SBR and EPDM composites for various times of vulcanization, which indicates the overall connectivity of conductive pathways within the rubber matrix. The CB concentration of 70 phr was selected to be above the percolation threshold based on our previously published data [3]. Higher conductivity values of the composites were observed with increasing vulcanization time, suggesting an effective connection of the conductive networks through the composite. Generally, the conductive rubber composite contains continuous paths of filler particles within the matrix, making the composite electrically more conductive. CB particles have a greater tendency to form conductive networks than other conductive fillers, due to their ability to form chain-like aggregates [23]. Therefore, the reason for the increase in conductivity with rising vulcanization time may consist in the higher crosslink density and consequently bigger CB aggregates. Thus, more stable conducting CB paths will be formed in the rubber matrix. Furthermore, as depicted in Figure 4, conductivity increases with rising frequency due to the growing importance of polarization effects [24].

The conductivity values differ for the SBR and EPDM composites. Higher conductivity of EPDM vulcanizates compared to SBR may consist in the lower affinity of CB particles with the EPDM matrix due to the more hydrophobic nature of the rubbery phase.

### 3.5. Conductivity Changes during Cyclic Mechanical Deformation

**Figure 5:**
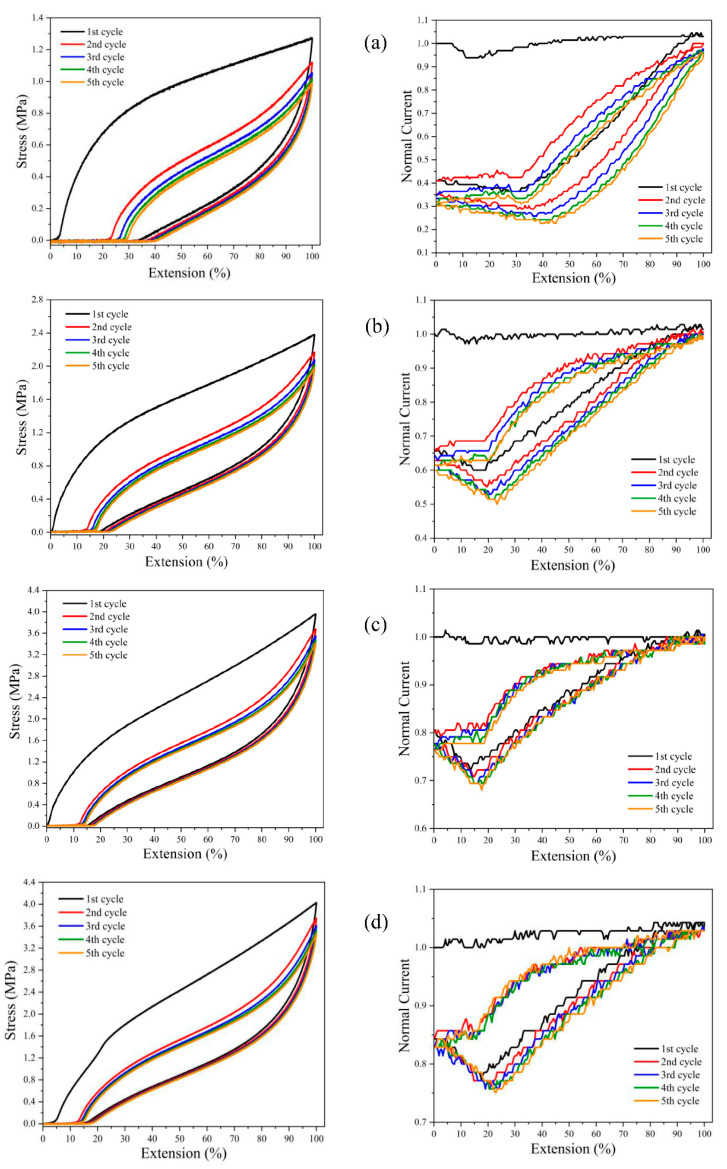
Dependence of stress (left) and normalized current (right) on deformation during five runs of cyclic deformation for SBR composites vulcanized at 150 °C for various times of (**a**) 3, (**b**) 8, (**c**) 30, and (**d**) 60 min.

**Figure 6:**
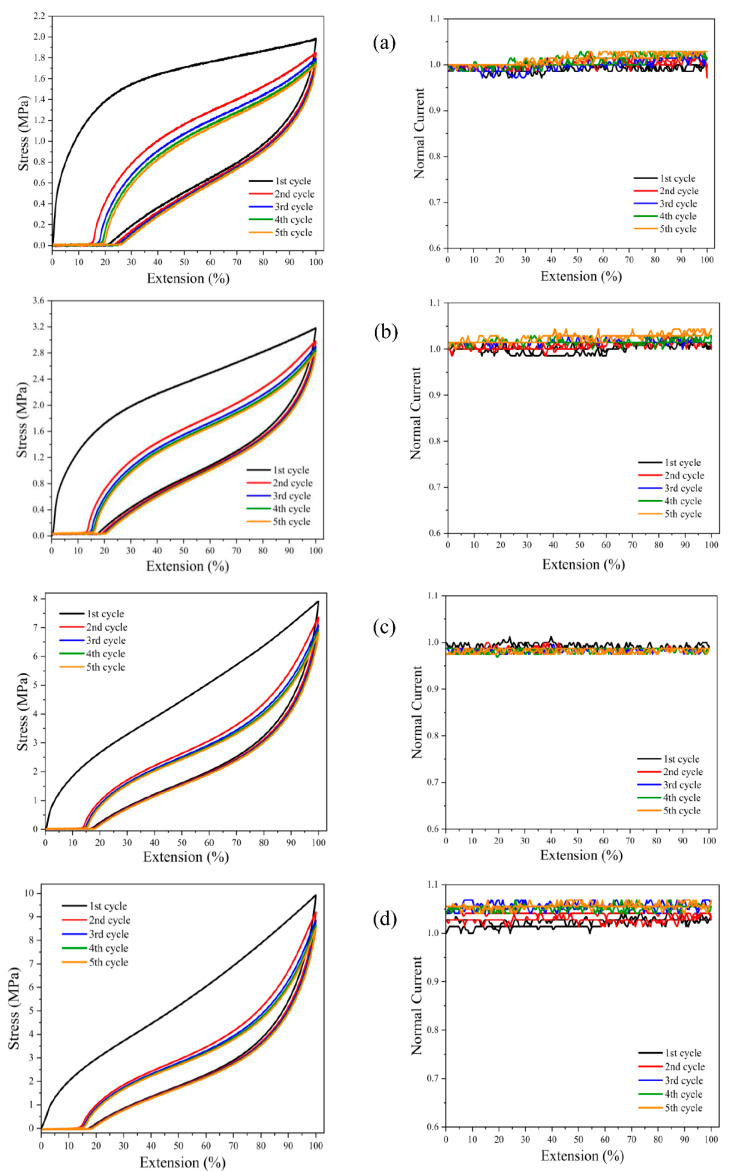
Dependence of stress (left) and normalized current (right) on deformation during five runs of cyclic deformation for EPDM composites vulcanized at 150 °C for various times of (**a**) 3, (**b**) 8, (**c**) 30, and (**d**) 60 min.

The courses of changes in electrical conductivity during five runs of cyclic deformations with an ultimate extension of 100% are shown in Figure 5 and Figure 6 for SBR and EPDM composites, respectively, vulcanized for various times. The standard shapes of stress-strain cycles with typical hysteresis can be seen for both types of composite, where the stress is considerably higher than the stress values during the decrease in deformation. For both SBR and EPDM composites, it is observed that the first cycle differs significantly from all other cycles, while all following dependences are nearly identical for the other cycles. The interesting deformation region belongs to deformations between 20% and 100%, where the inflection point on the stress-strain curve appears. The appearance of the inflex point was attributed to a process similar to the thermoplastics’ behavior around the yield point, leading to the beginning of the plastic flow of the material [3]. This behavior indicates that the rising stress in this region may correlate to certain extent with a formation of more stable CB networks being more resistant to external stress, resulting also in the reinforcement of the composite.

The same courses with a kind of “electrical hysteresis” are observed also for electrical conductivities of the SBR composite, unlike the EPDM composite. In the rising strain process, the current in the SBR composite is rising permanently, although not linearly, whereas in the EPDM composite, only a slight increase or even almost no change in current is observed. Higher conductivity during each consecutive deformation cycle translates into healing of minor defects in the conductive pathways or the formation of new conductive paths, due to the movement of rubber chains and the simultaneous movement of the filler particles which are ordering in the new conductive paths. It is known that mechanical deformation is a key factor affecting the changes in conductive paths, leading to significant destruction of the filler network [25]. However, a massive formation of new conductive paths in rubber [26], as well as in thermoplastics, e.g., polycaprolactone or thermoplastic starch [2,3,8,27], has been reported.

Concerning the relation of conductivity changes with the time of vulcanization, it is seen that the current values starting from the second cycle are increasing with rising vulcanization time in the SBR composite, while the values are nearly constant for all cycles in the EPDM composite. The increase in the current values of SBR vulcanizate could be attributed to the crosslink density increase, resulting in bigger CB aggregates and the presence of more stable conducting CB paths within the matrix. In case of the EPDM vulcanizate, which is capable of undergoing strain-induced crystallization [18], large CB aggregates form due to the more hydrophobic nature of the rubbery phase, and consequently the affinity of CB with the EPDM matrix is lower. Therefore, the conductive filler structure is strong enough to remain intact and maintain constant conductivity. However, mechanical deformation might lead to the decay of some pathways. The absolute values of the current at the starting point of the first cycle for SBR and EPDM composites vulcanized for various times are summarized in Table 6. These results also indicate that the conductivity at the beginning of the first cycle increases slightly with the increase in vulcanization time for all rubber composites investigated.

## 4. Conclusions

Changes in the electrical properties of flexible rubber-based conducting composites during cyclic mechanical deformation have been investigated in detail. This work provides novel insight into comparing two elastomeric matrices e.g., SBR and EPDM filled with highly conductive carbon black reinforcing filler to reveal rather delicate differences in the behavior of two matrices with slightly different affinity with the filler. Moreover, the effect of vulcanization time on the conductivity behavior of these matrices was evaluated. The higher vulcanization time resulted in a substantial increase in tensile strength and Young’s modulus, while a decrease in elongation at break was observed. The composites demonstrated an increase in the crosslink density determined by the decrease in the solvent uptake with rising vulcanization time, resulting also in higher static conductivity values of both types of composites and the T_g_ increase revealed by the DMTA results, indicating higher rigidity and lower molecular mobility. The EPDM vulcanizates showed higher static conductivity values compared to SBR vulcanizates, which is attributed to the lower affinity of CB particles with the EPDM matrix due to the more hydrophobic nature of the rubbery phase. In the rising strain period, the current in the SBR composite increased permanently, although not linearly, whereas, the EPDM composite showed a nearly constant current or a slight increase, indicating healing of the minor defects in the conductive network or the formation of new conductive pathways. For the SBR composite, the current value starting from the second cycle increased with rising vulcanization time in the SBR composite, while the current values were nearly constant for all cycles in the EPDM composite. We expect that the presented results have the potential to be considered in designing and constructing flexible conducting materials for engineering applications.

## Figures and Tables

**Table 1:** Properties of the carbon blacks (Vulcan N–234) used as filler.

Property	Amount
Iodine adsorption	120 mg/g
DBF adsorption	125 mL/100 g
Specific surface area	100–120 m^2^/g
pH	5.5–8.5
Particle diameter	20–25 nm

**Table 2:** Composition of rubber compounds in phr and their designation.

Component	Concentration (phr)
Rubber (SBR or EPDM) ^a^	100
CB	70
ZnO	3
Stearic acid	1
CBS	1
Sulfur	1.75

^a^ The samples were marked as SBR-X and EPDM-X, where X is the time of curing in minutes at 150 °C.

**Table 3:** Mechanical properties, including tensile strength, elongation at break, Young’s modulus, and moduli M100, M200, and M300 of the SBR and EPDM composites. The vulcanization time at 150 °C is shown in minutes as the number after code SBR- or EPDM-.

Sample Code	Tensile Strength (MPa)	Elongation at Break (%)	Young’s Modulus (MPa)	M 100 (MPa)	M 200 (MPa)	M 300 (MPa)
SBR-3	2.8 ± 0.4	240 ± 29	7.3 ± 0.4	1.8 ± 0.1	2.6 ± 0.2	n/a
SBR-8	16.1 ± 2.6	418 ± 16	4.8 ± 0.7	3.1 ± 0.2	6.5 ± 0.6	11.0 ± 1.2
SBR-30	26.5 ± 2.1	326 ± 17	10.7 ± 0.3	5.2 ± 0.1	13.3 ± 0.3	23.8 ± 0.6
SBR-60	22.8 ± 3.3	270 ± 28	11.4 ± 0.2	5.9 ± 0.1	15.0 ± 0.2	n/a
EPDM-3	4.0 ± 1.9	807 ± 146	14.8 ± 4.4	1.7 ± 0.3	2.0 ± 0.5	2.5 ± 0.8
EPDM-8	27.6 ± 2.6	398 ± 32	8.6 ± 0.4	5.1 ± 0.3	11.6 ± 0.8	20.0 ± 1.1
EPDM-30	26.0 ± 4.7	223 ± 36	14.3 ± 0.5	9.7 ± 0.4	23.2 ± 0.8	n/a
EPDM-60	20.4 ± 0.7	151 ± 2	16.3 ± 0.8	12.4 ± 0.9	n/a	n/a

**Table 4:** The solvent uptake and crosslink density of SBR and EPDM composites vulcanized for various times. The vulcanization time at 150 °C is shown in minutes as the number after code SBR- or EPDM-.

Sample Code	Solvent Uptake (%)	Crosslink Density *ν* (mol·m^−3^)
SBR-3	344 ± 20	236 ± 20
SBR-8	267 ± 5	361 ± 12
SBR-30	169 ± 2	779 ± 13
SBR-60	160 ± 2	850 ± 18
EPDM-3	371 ± 16	78 ± 9
EPDM-8	105 ± 9	1113 ± 140
EPDM-30	82 ± 1	1703 ± 16
EPDM-60	79 ± 2	1812 ± 60

**Table 5:** Dependence of glass transition temperature determined according to data from Figure 3 on crosslink density of SBR and EPDM composites vulcanized for various times. The vulcanization time at 150 °C is shown in minutes as the number after code SBR- or EPDM-.

Sample Code	Glass Transition T_g_ (°C)	Crosslink Density *ν* (mol·m^−3^)
SBR-3	−33.1	236 ± 20
SBR-8	−30.3	361 ± 12
SBR-30	−28.6	779 ± 13
SBR-60	−25.1	850 ± 18
EPDM-3	−25.3	78 ± 9
EPDM-8	−26.9	1113 ± 140
EPDM-30	−24.9	1703 ± 16
EPDM-60	−21.1	1812 ± 60

**Table 6:** The values of the current measured before starting the cyclic deformation experiments for SBR and EPDM composites vulcanized for various times. The vulcanization time at 150 °C is shown in minutes as the number after code SBR- or EPDM-.

Sample Code	Current before Starting of Cyclic Deformation (μA)
SBR-3	0.66 ± 0.00
SBR-8	0.70 ± 0.01
SBR-30	0.72 ± 0.00
SBR-60	0.70 ± 0.01
EPDM-3	0.66 ± 0.02
EPDM-8	0.69 ± 0.01
EPDM-30	0.79 ± 0.04
EPDM-60	0.73 ± 0.01
